# Management of upper gastrointestinal bleeding in emergency departments, from bleeding symptoms to diagnosis: a prospective, multicenter, observational study

**DOI:** 10.1186/s13049-017-0425-6

**Published:** 2017-08-14

**Authors:** Pierre-Clément Thiebaud, Youri Yordanov, Jacques-Emmanuel Galimard, Pierre-Alexis Raynal, Sébastien Beaune, Laurent Jacquin, François-Xavier Ageron, Dominique Pateron, the Initiatives de Recherche aux Urgences Group

**Affiliations:** 1 Service des Urgences - Hôpital Saint Antoine, Assistance Publique–Hôpitaux de Paris (APHP), Paris, France; 2 Initiatives de Recherche aux Urgences, SFMU, French Society of of Emergency Medicine, Paris, France; 3 Sorbonne Universités, UPMC Paris Univ-06, Paris, France; 4 INSERM, U1153, Paris, France; 5 INSERM U1153, Statistic and Epidemiologic Research Center Sorbonne Paris Cité (CRESS), ECSTRA Team, Saint-Louis Hospital, Paris, France; 6 Emergency department, CHU Ambroise Paré, Assistance Publique Hôpitaux de Paris, Boulogne-Billancourt, France; 7 Emergency department, Hôpital Edouard Herriot, Hospices Civils de Lyon, Lyon, France; 8 000 0004 0639 3167 Emergency department, Centre Hospitalier Annecy Genevois, Pringy Cedex, Metz-Tessy, France

**Keywords:** Gastrointestinal bleeding, Hematemesis, Melena, Endoscopy, Emergency department

## Abstract

**Background:**

Upper gastrointestinal bleeding (UGB) is common in emergency departments (EDs) and can be caused by many eso-gastro-duodenal lesions. Most available epidemiological data and data on the management of UGB comes from specialized departments (intensive care units or gastroenterology departments), but little is known from the ED perspective.

We aimed to determine the distribution of symptoms revealing UGB in EDs and the hemorrhagic lesions identified by endoscopy. We also describe the characteristics of patients consulting for UGB, UGB management in the ED and patients outcomes.

**Method:**

This was a prospective, observational, multicenter study covering 4 consecutive days in November 2013. Participating EDs were part of the Initiatives de Recherche aux Urgences network coordinated by the French Society of Emergency Medicine. All patients with suspected UGB in these EDs were included.

**Results:**

In total, 110 EDs participated, including 194 patients with suspected UGB (median age 66 years [Q1-Q3: 51-81]). Overall, 104 patients (54%) had hematemesis and 75 (39%) melena. Endoscopy revealed lesions in 121 patients, mainly gastroduodenal ulcer or ulcerations (41%) or bleeding lesions due to portal hypertension (20%). The final diagnosis of UGB was reversed by endoscopy in only 3% of cases. Overall, 67 patients (35%) had at least one severity sign. Twenty-one patients died (11%); 40 (21%) were hospitalized in intensive care units and 126 (65%) in medicine departments; 28 (14%) were outpatients. Mortality was higher among patients with clinical and biological severity signs.

**Conclusion:**

Most of the UGB cases in EDs are revealed by hematemesis. The emergency physician diagnosis of UGB is rarely challenged by the endoscopic findings.

**Electronic supplementary material:**

The online version of this article (doi:10.1186/s13049-017-0425-6) contains supplementary material, which is available to authorized users.

## Background

Upper gastrointestinal bleeding (UGB) is a common emergency, with a variety of eso-gastro-duodenal symptoms as hematemesis, melena, less often hematochezia or anemia. It can be caused by various potentially serious lesions, as peptic ulcers or varices [1]. UGB annual incidence tends to decrease, influenced by the latest therapeutical developments in the management of peptic ulcers or the prevention of portal hypertension complications [2]. But it remains relatively high because of the widespread use of non-steroidal anti-inflammatory drugs (NSAIDS) and anti-thrombotic agents [3, 4]. Current cases of UGB occur in patients older than previously described [4–6]. Several recommendations regarding UGB management have been published, including therapeutic interventions initiated by the emergency physicians. The implementation of these recommendations could affect patients’ prognosis [2, 7, 8].

Very few studies have been conducted in the emergency setting (hospital and pre-hospital settings) to describe UGB epidemiology and management [9]. Existing epidemiological data usually concern UGB in patients hospitalized in gastroenterology, intensive care units or after an endoscopy [10–13]. But these patients represent only a portion of all those with UGB seen in emergency departments (EDs). Specifically targeting UGB patients presenting in EDs could give us a more comprehensive epidemiological description.

Therefore, we aimed at describing the distribution of symptoms revealing UGB in EDs and the hemorrhagic lesions identified by endoscopy. Our secondary objectives were to describe the epidemiological characteristics and management of patients with UGB, including outcomes.

## Methods

This prospective, observational, multicenter study was conducted over 4 consecutive days in November 2013. We included the 110 EDs, 17 with a prehospital medical unit, that were part of a network of clinical research (Initiatives de Recherche aux Urgences [IRU]) coordinated by the French Society of Emergency Medicine [SFMU]. The IRU correspondent for each ED was responsible for patients’ inclusion and data extraction. The STROBE recommendations for reporting of observational studies were followed [14].

### Patients

All patients with suspected UGB, hematemesis with or without melena, melena without hematemesis, hematochezia or other symptoms (acute anemia, hemorrhagic shock or syncope) suggestive of hemorrhage, who were seen in one of the participating pre-hospital or hospital EDs were included by the emergency physicians of the participating departments. Exclusion criteria were patients aged less than 18 years old, refusal to participate or already included in the study once. For each patient, the following data were collected: type of first contact with an emergency medicine structure (hospital or pre-hospital), age, sex, first symptoms (hematemesis, melena, other), medical history (cirrhosis, ulcer and other comorbidities), and NSAIDS and/or antithrombotic treatment. In light of existing epidemiological data, showing an annual incidence of high digestive bleeding of 100 to 150/100,000 inhabitants [5], the number of centres in the IRU network and the duration of the study of 4 days, we expected to include 150 to 300 cases of UGB. We estimated the prevalence of UGB with 2013 data from the French Emergency Survey (FES) and the National Institute of Statistics and Economic Studies (INSEE) data.

### Assessment of disease severity

Data for the initial clinical items related to severity were collected, including signs related to blood loss (heart rate > 100 bpm, systolic blood pressure < 90 mmHg, marbling, altered mental status) and haemoglobinemia (> 10, 7–10 and <7 g/dL hemoglobin level).

### Management and treatment

The following therapeutic measures, done in the ED, were collected: placement of a nasogastric tube; fluid administration; transfusion; and use of proton pump inhibitors, vasopressors (somatostatin, octreotide, terlipressin), catecholamines (adrenaline, norepinephrine), anticoagulation reversal and antibiotics. The use of erythromycin before endoscopy was also noted. Endoscopy data concerning bleeding lesions and hemostasis procedures were noted, as were the performance of any imaging test (ultrasonography, CT). Patients outcome (hospitalised or outpatient), final diagnosis and hospital deaths were also collected.

### Statistical analysis

Continuous variables are presented as median, first and third quartile (Q1-Q3) and were compared using the Wilcoxon rank sum test. Categorical variables are expressed as number and percentage. They were compared using Fisher’s exact test. Statistical analyses were two-tailed, and a *p* value less than 0.05 was considered significant. Analyses were performed using R statistical software, version 3.1.3 (www.r-project.org).

### Ethics

The study was approved by the institutional review board (IRB) (Comité de protection des personnes, Ile de France XI, Paris, France) and the Advisory Committee on Information Processing in Material Research in the Field of Health (CCTIRS). Patients, or their next of kin, were informed that a study was being led and that their data might be used. They could refuse being included.

## Results

**Fig. 1:**
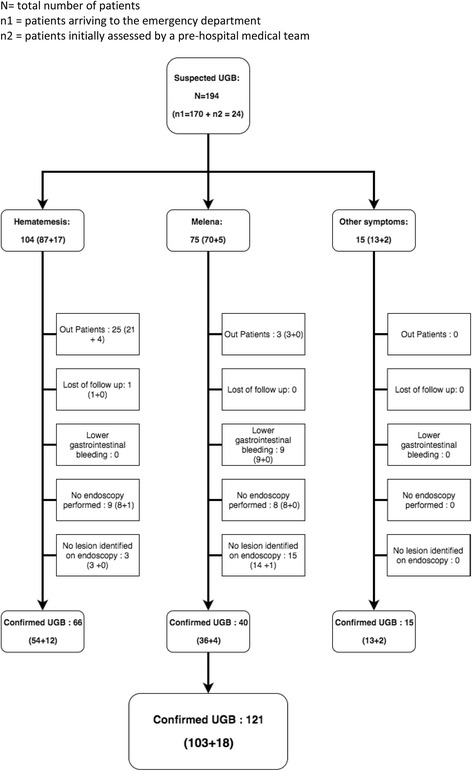
Flow of patients from the first symptoms of suspected upper gastrointestinal bleeding (UGB) to endoscopy diagnosis. N = total number of patients. n1 = patients arriving to the emergency department. n2 = patients initially assessed by a pre-hospital medical team

During the study, we have included 194 patients with suspected UGB, No patients declined participation, no patients were excluded due to multiple inclusions, and two patients younger than 18 years old were not included in the study. The participating EDs received 46.190 visits during the study period and UGB was suspected in 0.42% of the situations. Thus, the estimated incidence of UGB in France, in 2013, was 122/100,000 inhabitants. Out of the 194 included patients, 24 received initial prehospital medical care (12%), median age was 66 years [Q1-Q3 51-81] and 105 (54%) were male. Overall, 104 (54%) had hematemesis and 75 melena (39%). For 15 patients (8%), the suspicion was based on other symptoms (Table 1). Bleeding externalization was observed during the ED stay of a 102 patients (53%). The flow from first symptoms to endoscopy diagnosis is reported in Fig. 1. In total, 148 patients (76%) underwent endoscopy during their hospital stay, out of which 44 (23%) during the ED stay: 9/44 (20%) received erythromycin and 12/44 (27%) a hemostatic procedure. Endoscopy confirmed the diagnosis and revealed lesion explaining UGB in 121/148 patients (82%) (Table 2). Gastroduodenal ulcer (44/148 patients, 30%) was the most frequent lesion followed by variceal bleeding (30/148, 20%) and gastritis (16/148, 11%). In 22/148 patients (15%), no lesion was found. In 5/148 (3%) the diagnosis of UGB was overturned, with lower gastrointestinal bleeding finally diagnosed.

**Table 1:** General characteristics and treatments of patients presenting an upper gastrointestinal bleeding (UGB)

		Arrival at the ED *n* = 170	Pre-hospital management *n* = 24	Total *n* = 194	*P* value
Age (median [Q1–Q3])^a^		66 [49–82]	71 [57.5–75.5]	66 [51–81]	0.71
Sex (men; n, %)		88 (52)	17 (71)	105 (54)	0.085
Initial symptoms suggesting UGB, n (%)	Hematemesis	87 (51)	17 (71)	104 (54)	0.096
	Melena	70 (41)	5 (21)	75 (39)	
	Hematochezia	6 (4)	2 (8)	8 (4)	
	Other	7 (4)	0 (0)	7 (4)	
Medical history and medication, n (%)^b^		*n* = 143	*n* = 20	*n* = 163	
Known cirrhosis		31 (22)	4 (20)	35 (21)	1.
Known ulcer		33 (23)	5 (25)	38 (23)	0.78
Non-steroid anti-inflammatory drugs		10 (7)	2 (10)	12 (7)	0.64
Antithrombotic agents		54 (38)	8 (40)	62 (38)	1.
Comorbidity		57 (40)	5 (25)	62 (38)	0.25
Exteriorized bleeding in the ED, n (%)^c^		86 (51)	16 (67)	102 (53)	0.19
Clinical features of severity, n (%)^d^		*n* = 166	*n* = 22	*n* = 188	
Heart rate > 100 bpm		36 (22)	12 (55)	48 (26)	0.003
Systolic arterial pressure < 90 mmHg		27 (16)	8 (36)	35 (19)	0.037
Marbling		5 (3)	3 (14)	8 (4)	0.053
Altered mental status		5 (3)	4 (18)	9 (5)	0.012
Hemoglobin level, n (%)^e^	< 7 g/dL	34 (20)	5 (24)	39 (21)	0.77
Treatments in ED, n (%)		*n* = 170	*n* = 24	*n* = 194	
Nasogastric tube		15 (9)	4 (17)	19 (10)	0.26
Fluid administration		55 (32)	12 (50)	67 (35)	0.11
Transfusion		71 (42)	1 (4)	72 (37)	0.0002
Proton pump inhibitors		132 (78)	8 (33)	140 (72)	< 0.0001
Vasopressors		33 (19)	3 (13)	36 (19)	0.58
Catecholamines		2 (1)	2 (8)	4 (2)	0.075
Antibiotics^f^ (excluding erythromycin)		8 (5)	---	---	---
Vitamin K antagonist reversal		18 (11)	1 (4)	19 (10)	0.048
Erythromycin		16 (9)	1 (4)	17 (9)	0.70

^a^for 193 of 194 patients

^b^for 163 of 194 patients

^c^for 192 of 194 patients

^d^for 188 of 194 patients

^e^for 189 of 194 patients

^f^for 170 of 194 patients

**Table 2:** Final diagnosis

	Final diagnosis	n (%)
Endoscopy performed, *n* = 148 (76%)	Ulcers	44 (30)
	Gastritis	16 (11)
	Variceal bleeding	30 (20)
	Esophagitis	12 (8)
	Mallory-Weiss tear	7 (5)
	Cancer	12 (8)
	Lower gastrointestinal bleeding	5 (3)
	None	22 (15)^a^
No endoscopy performed, *n* = 46 (24%)	Lower gastrointestinal bleeding	4 (9)
	None	42 (91)^b^

^a^5 outpatients included

^b^23 outpatients included

Overall, 67/194 patients (35%) had at least one severity sign: 48 (26%) tachycardia, 35 (19%) hypotension, some could present several severity sign (Table 1). Thirty-nine patients (21%) had a hemoglobin level < 7 g/dL and 72 (37%) underwent transfusion. Data regarding type of UGB management are presented in Table 1. Thirty-five patients (21%) had a known cirrhosis. Clinical characteristics and outcomes depending on presence or not of cirrhosis are presented in Table 3. Patients with a history of cirrhosis were more often younger and males than those without cirrhosis. They presented with a significantly higher proportion of severity signs, as heart rate > 100 bpm (*p* = 0.006), marbling (*p* = 0.031) or an altered mental status (*p* = 0.015). A total of 21 patients (11%) died, of which 3 during the pre-hospital phase; 40 (21%) were hospitalized in intensive care units, 126 (65%) in medicine departments and 28 (14%) were outpatients.

**Table 3:** Patients characteristics depending on their cirrhosis history

		With cirrhosis *n* = 35	Without cirrhosis *n* = 128	*P* value
Age (median [Q1–Q3])		56 [50–67.5]	73 [56–83]	0.0006
Sex (men; n, %)		26 (74%)	69 (54%)	0.034
Initial symptoms suggesting UGB, n (%)	Hematemesis	24 (69)	60 (47)	0.005
	Melena	7 (20)	60 (47)	
	Hematochezia	1 (3)	6 (5)	
	Other	2 (2)	3 (9)	
Exteriorized bleeding in the ED, n (%)^a^		21 (62)	69 (54)	0.56
Clinical features of severity, n (%)^b^		*n* = 32	*n* = 127	
Heart rate > 100 bpm		17 (53)	26 (20)	0.0006
Systolic arterial pressure < 90 mmHg		9 (28)	22 (17)	0.21
Marbling		4 (13)	3 (2)	0.031
Altered mental status		4 (13)	2 (2)	0.015
Hemoglobin level, n (%)^b^	< 7 g/dL	10 (29)	26 (21)	0.35
Upper endoscopy in ED, n (%)^c^		29 (26)	9 (29)	0.82
Hemostatic procedure^d^		5 (17)	5 (5)	0.036
Treatments in ED, n (%)		*n* = 35	*n* = 128	
Nasogastric tube		5 (14)	12 (9)	0.37
Fluid administration		20 (57)	38 (30)	0.005
Transfusion		16 (46)	50 (39)	0.56
Proton pump inhibitors		28 (80)	93 (73)	0.51
Vasopressors		23 (66)	10 (8)	<0.0001
Catecholamines		2 (6)	1 (1)	0.12
Antibiotics^c^ (excluding erythromycin)		4 (13)	2 (2)	0.020
Vitamin K antagonist reversal		0	19 (15)	0.027
Erythromycin		6 (17)	9 (7)	0.09
Final diagnosis^e^ (hospitalized patients), n (%)	Ulcer	2 (6)	38 (37)	<0.0001
	Gastritis	3 (9)	12 (12)	
	Variceal bleeding	25 (74)	1 (1)	
	Esophagitis	2 (6)	9 (9)	
	Mallory-Weiss tear	0	6 (6)	
	Cancer	0	12 (12)	
	Lower GI bleeding	0	6 (6)	
	None	2 (6)	19 (18)	

^a^for 161 of 163 patients

^b^for 159 of 163 patients

^c^for 143 of 163 patients

^d^for 141 of 163 patients

^e^for 137 of 163 patients

Mortality was significantly higher for hypotensive patients (*p* = 0.004), with marbling (*p* = 0.042) or altered mental status (*p* = 0.0008). Exteriorized bleeding in the ED was also associated to a higher mortality rate (*p* = 0.035). Deceased patients received more frequently vasopressors (*p* = 0.0009) or catecholamines (*p* = 0.004). Although not significant, there seem to be a trend between mortality and cirrhosis (*p* = 0.071) and transfusion (*p* = 0.057) (Table 4).

**Table 4:** Patients characteristics depending on their survival status

		Survivors *n* = 172^a^	Dead *n* = 21^a^	*P* value
Age (median [Q1–Q3])^b^		66.5 [49–81]	66 [57–83]	0.46
Sex (men; n, %)		91 (53)	13 (62)	0.49
Initial symptoms suggesting UGB, n (%)	Hematemesis	90 (52)	13 (62)	0.87
	Melena	67 (39)	8 (38)	
	Hematochezia	8 (5)	0 (0)	
	Other	7 (4)	0 (0)	
Medical history and medication, n (%)^c^		*n* = 144	*n* = 18	
Known cirrhosis		28 (19)	7 (39)	0.071
Known ulcer		35 (24)	3 (17)	0.57
Non-steroid anti-inflammatory drugs		54 (38)	8 (44)	0.61
Antithrombotic agents		58 (40)	4 (22)	0.20
Comorbidity		12 (8)	0 (0)	0.36
Exteriorized bleeding in the ED, n (%)^d^		85 (50)	16 (76)	0.035
Clinical features of severity, n (%)^e^		*n* = 167	*n* = 20	
Heart rate > 100 bpm		39 (23)	8 (40)	0.11
Systolic arterial pressure < 90 mmHg		26 (16)	9 (45)	0.004
Marbling		5 (3)	3 (15)	0.042
Altered mental status		4 (2)	5 (25)	0.0008
Hemoglobin level n (%)^f^	< 7 g/dL	34 (20)	5 (24)	0.55
Upper endoscopy in ED, n (%)^g^		39 (25)	5 (31)	0.57
Hemostatic procedure^h^		11 (7)	1 (7)	1.00
Treatments in ED, n (%)		*n* = 172	*n* = 21	
Nasogastric tube		18 (10)	1 (5)	0.70
Fluid administration		58 (34)	8 (38)	0.81
Transfusion		60 (35)	12 (57)	0.057
Proton pump inhibitors		125 (73)	14 (67)	0.61
Vasopressors		25 (15)	10 (48)	0.0009
Catecholamines		1 (1)	3 (14)	0.004
Antibiotics^g^ (excluding erythromycin)		7 (5)	1 (6)	0.56
Vitamin K antagonist reversal		16 (9)	3 (14)	0.44
Erythromycin		15 (9)	2 (10)	1.00
Final diagnosis, n (%)^i^ (hospitalized patients)	Ulcer	41 (29)	3 (19)	0.31
	Gastritis	15 (11)	1 (6)	
	Variceal bleeding	23 (17)	7 (44)	
	Esophagitis	12 (9)	0 (0)	
	Mallory-Weiss tear	7 (5)	0 (0)	
	Cancer	10 (7)	2 (13)	
	Lower GI bleeding	8 (6)	1 (6)	
	None	23 (17)	2 (13)	

^a^1 missing data

^b^for 192 of 193 patients

^c^for 162 of 193 patients

^d^ for 191 of 193 patients

^e^for 187 of 193 patients

^f^for 188 of 193 patients

^g^for 169 of 193 patients

^h^for 167 of 193 patients

^i^for 155 of 193 patients

## Discussion

The distribution of symptoms for suspected UGB is poorly known, especially in EDs, even though most cases of UGB (80–90%) are managed in EDs [11, 15, 16]. Our multicentric, prospective study performed over a short period (4 days) in French EDs found that for more than half of the patients (54%), the UGB was revealed by hematemesis. Endoscopy revealed a lesion in about 80% of patients. The final diagnosis of UGB was reversed for only 3% of patients. More than one third of patients had at least one severity sign; about 20% had hypotension, < 7 g/dL hemoglobin level, and a known cirrhosis. In all, 11% of patients died; initial hypotension, marbling or altered mental status were significantly linked to mortality.

Our proportion of patients presenting with hematemesis (54%) is close to literature data (42–61%) for UGB managed in gastroenterology departments, intensive care units or by emergency endoscopy [3, 9, 17, 18]. In a study of 1140 emergency and ambulatory care patients with UGB of ulcerative origin, the proportion of melena was higher than in our study (52 vs 39%), with 40% of patients having hematemesis and 8% anemia without exteriorized bleeding [19]. In our study, bleeding lesions were diagnosed by endoscopy in 80% of cases. One quarter of patients had no endoscopy during hospitalization, often because of the low severity among outpatients and more rarely (4 cases) because the patient died before endoscopy could be performed. This might have an impact on the distribution of the causes of UGB. In about 15% of patients, endoscopic diagnosis was missing, which is comparable to previously published studies [5, 20, 21]. The prevalence of 41% of lesions with an ulcerative origin (ulcer disease or complicated ulcerations) is close to data (28-67%) from studies including UGB cases from endoscopy examination [5, 21]. The 20% of bleeding lesions due to portal hypertension was associated to the high prevalence of cirrhosis in our population [22]. More than a quarter of patients with cirrhosis presented bleeding from other causes than cirrhosis. This can be a strong argument in favor of the use of proton pump inhibitors before endoscopy in this subset of patients, as it’s recommended [7],. In our study, the diagnosis of UGB in the ED was rarely challenged by subsequent explorations (3% of cases).

The UGB incidence estimated from our study favours the completeness of data for our included patients. Epidemiological reviews show an annual incidence of UGB of 50 to 150/100,000 inhabitants [5] and the main French study showed an annual incidence of 146/100,000 [1]. The median age of UGB and proportion of patients older than 80 years is similar to that observed in the most recent studies and seems higher than that observed 10 years ago, with a significant proportion of patients on anti-thrombotic therapy [3, 4]. The proportion of patients with UGB who were older than 75 years was 27% in 1996 [23] but 37% in our study. Two studies [4, 6] confirmed an increase in the ageing of the population with UGB, with average age 57, 59, 63 and 66 years in 1986-1987, 1995, 2000-2001, and 2005, respectively. As in our study, the literature shows a male predominance, with a sex ratio between 1.3 and 2 [1, 20, 23], but the proportion of women with UGB is increasing [24].

The observed severity of disease in our patients was similar to epidemiological studies, finding mortality between 3 and 14% [4, 5, 17, 21]. Our level of mortality (11%) might seem relatively high for patients with UGB in EDs [5], possibly because we included all patients with suspected bleeding, including those with a history of cirrhosis that appeared more severe, and critically ill patients that were initially managed in the pre-hospital setting. The main factors associated with mortality found in the literature are ageing, co-morbidities (including cirrhosis), signs of severity, an initial low blood pressure, hematemesis and low hemoglobin level [4, 5, 20, 21]. We found a significant association between mortality and signs of severity (low blood pressure, marbling, altered mental status), exteriorized bleeding in the ED or therapeutical interventions as the use of catecholamines or vasoactive agents. There appear to be a trend toward an association with history of cirrhosis and need to transfusion, although not significant, which could be due to a lack of statistical power of our study. When looking at published literature, mortality was higher in patient with a history of cirrhosis [10, 23], with variceal bleeding [17, 20] but also for these patients with a bleeding ulcer [21]. Use of anti-thrombotic agents, a known risk factor of digestive hemorrhage [21], was frequent in our population but did not predict mortality.

Management of UGB in our cohort of patients shows an evolution of practices, possibly influenced by recommendations [2, 7, 8]. Proton pump inhibitors were used for three-quarters of our patients, and their administration is now recommended as soon as possible without waiting for endoscopy [7]. Most patients with cirrhosis received vasopressor treatment in the first 24 h [2]. The number of transfusions suggests that the policy of restricting transfusion is not yet followed [25]. Only a few patients received nasogastric tubes (10%). Several studies indicated that nasogastric tube placement does not confirm the upper origin of a gastrointestinal bleeding [26, 27] and recommendations remain unclear on this topic. Erythromycin perfusion before endoscopy is rarely used [28]. This practice, although validated by several studies, is not shared by some international recommendations [21]. The use of antibiotics in patients with cirrhosis remains low despite recommendations on this topic [29]. Outpatient care concerned only 14% of our patients, which is less than in studies using a severity score [30]. The use of these scores would probably increase the proportion of outpatients.

## Limitations

One main limitation of our study is the risk of selection. The departments participating in the study are a subset of the 600 French EDs, that are particularly interested in clinical research. Patients presenting at these EDs might not be representative of the general population. The IRU includes community and university hospitals, so this risk was deemed acceptable. Another limitation can be due to the short inclusion period of 4 days that might not perfectly reflect the distribution of the causes of upper GI bleeding. There’s also a possibility of under or overestimation of UGB incidence, due possible natural variations in the number of patients presenting with UGB in EDs. The third limitation is the absence of precise quantitative data. Each local investigator, when including patients, had to choose between various categories (e.g., tachycardia >100 bpm, hemoglobin level > 10, 7–10 and <7 g/dL) to simplify data extraction sheets and ensure data quality and comprehensiveness, at the expense of severity score precision. The number of inclusions (194 patients) limits the statistical power of the study, especially for prognostic factors. Moreover, the number of deceased patients did not allow us to perform a robust multivariate analysis.

## Conclusions

Most of the UGB cases in EDs are revealed by hematemesis. The emergency physician diagnosis of UGB is rarely challenged by the endoscopic findings. Epidemiological data for patients with UGB managed in the emergency departments are similar to the patients treated in gastroenterology departments and/or in intensive care units. More than one third of UGB patients are more than 75 years old.

## Additional file

Additional file 1: Appendix 1. Members of the Initiatives de Recherche aux Urgences network. (DOCX 16 kb)

## References

[1] Czernichow P, Hochain P, Nousbaum JB, Raymond JM, Rudelli A, Dupas JL (2000). Epidemiology and course of acute upper gastro-intestinal haemorrhage in four French geographical areas. Eur J Gastroenterol Hepatol.

[2] de Franchis R, Faculty BVI (2015). Expanding consensus in portal hypertension: report of the Baveno VI consensus workshop: stratifying risk and individualizing care for portal hypertension. J Hepatol.

[3] Hreinsson JP, Kalaitzakis E, Gudmundsson S, Björnsson ES (2013). Upper gastrointestinal bleeding: incidence, etiology and outcomes in a population-based setting. Scand J Gastroenterol.

[4] Thomopoulos KC, Vagenas KA, Vagianos CE, Margaritis VG, Blikas AP, Katsakoulis EC (2004). Changes in aetiology and clinical outcome of acute upper gastrointestinal bleeding during the last 15 years. Eur J Gastroenterol Hepatol.

[5] van Leerdam ME (2008). Epidemiology of acute upper gastrointestinal bleeding. Best Pract Res Clin Gastroenterol.

[6] Theocharis GJ, Thomopoulos KC, Sakellaropoulos G, Katsakoulis E, Nikolopoulou V (2008). Changing trends in the epidemiology and clinical outcome of acute upper gastrointestinal bleeding in a defined geographical area in Greece. J Clin Gastroenterol.

[7] Osman D, Djibré M, Da Silva D, Goulenok C, group of experts (2012). Management by the intensivist of gastrointestinal bleeding in adults and children. Ann Intensive Care.

[8] Barkun AN, Bardou M, Kuipers EJ, Sung J, Hunt RH, Martel M (2010). International consensus recommendations on the management of patients with nonvariceal upper gastrointestinal bleeding. Ann Intern Med.

[9] Chassaignon C, Letoumelin P, Pateron D, Group HD 2000 (2003). Upper gastrointestinal haemorrhage in emergency Departments in France: causes and management. Eur J Emerg Med Off J Eur Soc Emerg Med.

[10] van Leerdam ME, Vreeburg EM, Rauws E a J, Geraedts A a M, Tijssen JGP, Reitsma JB (2003). Acute upper GI bleeding: did anything change? Time trend analysis of incidence and outcome of acute upper GI bleeding between 1993/1994 and 2000. Am J Gastroenterol.

[11] Vreeburg EM, Snel P, de Bruijne JW, Bartelsman JF, Rauws EA, Tytgat GN (1997). Acute upper gastrointestinal bleeding in the Amsterdam area: incidence, diagnosis, and clinical outcome. Am J Gastroenterol.

[12] Longstreth GF (1995). Epidemiology of hospitalization for acute upper gastrointestinal hemorrhage: a population-based study. Am J Gastroenterol.

[13] Blatchford O, Davidson LA, Murray WR, Blatchford M, Pell J (1997). Acute upper gastrointestinal haemorrhage in west of Scotland: case ascertainment study. BMJ.

[14] von Elm E, Altman DG, Egger M, Pocock SJ, Gøtzsche PC, Vandenbroucke JP (2007). The strengthening the reporting of observational studies in epidemiology (STROBE) statement: guidelines for reporting observational studies. Lancet Lond Engl.

[15] Rockall TA, Logan RF, Devlin HB, Northfield TC (1995). Incidence of and mortality from acute upper gastrointestinal haemorrhage in the United Kingdom. Steering committee and members of the National Audit of acute upper gastrointestinal haemorrhage. BMJ.

[16] Paspatis GA, Matrella E, Kapsoritakis A, Leontithis C, Papanikolaou N, Chlouverakis GJ (2000). An epidemiological study of acute upper gastrointestinal bleeding in Crete, Greece. Eur J Gastroenterol Hepatol.

[17] Kim JJ, Sheibani S, Park S, Buxbaum J, Laine L (2014). Causes of bleeding and outcomes in patients hospitalized with upper gastrointestinal bleeding. J Clin Gastroenterol.

[18] Lanas A, Aabakken L, Fonseca J, Mungan ZA, Papatheodoridis GV, Piessevaux H (2011). Clinical predictors of poor outcomes among patients with nonvariceal upper gastrointestinal bleeding in Europe. Aliment Pharmacol Ther.

[19] Zeitoun J-D, Rosa-Hézode I, Chryssostalis A, Nalet B, Bour B, Arpurt J-P (2012). Epidemiology and adherence to guidelines on the management of bleeding peptic ulcer: a prospective multicenter observational study in 1140 patients. Clin Res Hepatol Gastroenterol.

[20] Nahon S, Hagège H, Latrive JP, Rosa I, Nalet B, Bour B (2012). Epidemiological and prognostic factors involved in upper gastrointestinal bleeding: results of a French prospective multicenter study. Endoscopy.

[21] Rotondano G (2014). Epidemiology and diagnosis of acute nonvariceal upper gastrointestinal bleeding. Gastroenterol Clin N Am.

[22] Garcia-Tsao G, Bosch J (2010). Management of varices and variceal hemorrhage in cirrhosis. N Engl J Med.

[23] Di Fiore F, Lecleire S, Merle V, Hervé S, Duhamel C, Dupas J-L (2005). Changes in characteristics and outcome of acute upper gastrointestinal haemorrhage: a comparison of epidemiology and practices between 1996 and 2000 in a multicentre French study. Eur J Gastroenterol Hepatol.

[24] Loperfido S, Baldo V, Piovesana E, Bellina L, Rossi K, Groppo M (2009). Changing trends in acute upper-GI bleeding: a population-based study. Gastrointest Endosc.

[25] Villanueva C, Colomo A, Bosch A, Concepción M, Hernandez-Gea V, Aracil C (2013). Transfusion strategies for acute upper gastrointestinal bleeding. N Engl J Med.

[26] Cuellar RE, Gavaler JS, Alexander JA, Brouillette DE, Chien MC, Yoo YK (1990). Gastrointestinal tract hemorrhage. The value of a nasogastric aspirate. Arch Intern Med.

[27] Kessel B, Olsha O, Younis A, Daskal Y, Granovsky E, Alfici R (2016). Evaluation of nasogastric tubes to enable differentiation between upper and lower gastrointestinal bleeding in unselected patients with melena. Eur J Emerg Med Off J Eur Soc Emerg Med.

[28] Pateron D, Vicaut E, Debuc E, Sahraoui K, Carbonell N, Bobbia X (2011). Erythromycin infusion or gastric lavage for upper gastrointestinal bleeding: a multicenter randomized controlled trial. Ann Emerg Med.

[29] Chavez-Tapia NC, Barrientos-Gutierrez T, Tellez-Avila FI, Soares-Weiser K, Uribe M. Antibiotic prophylaxis for cirrhotic patients with upper gastrointestinal bleeding. Cochrane Database Syst Rev. 2010;(9):CD002907. 10.1002/14651858.CD002907.pub2 PMC7138054 20824832

[30] Longstreth GF, Feitelberg SP (1995). Outpatient care of selected patients with acute non-variceal upper gastrointestinal haemorrhage. Lancet Lond Engl.

