# Supplementary material for: Management of upper gastrointestinal bleeding in emergency departments, from bleeding symptoms to diagnosis: a prospective, multicenter, observational study
Source: Scand J Trauma Resusc Emerg Med. 2017 Aug 14;25:78. doi: 10.1186/s13049-017-0425-6 (PMC5557479; doi:10.1186/s13049-017-0425-6)
Supplement: Additional file 1: Appendix 1. — Members of the Initiatives de Recherche aux Urgences network. (DOCX 16 kb) [file 13049_2017_425_MOESM1_ESM.docx]

**Appendix 1: Members of the Initiatives de Recherche aux Urgences network participating in the study.**

ABDELLI Leïla, Tourcoing. ANDREGNETTE Paul, Lens. ANDRIANJAFY Héry, Longjumeau. ARNAUD Aurélie, Montluçon. AUFFRET Yannick, Quimper. BARONE Florent-Alexis, Beauvais. BEBIEN Laurent, Sète. BENABBAS Samir, Gisors. BENDIB Narjess, Jossigny. BEREAU Justine, Bordeaux. BERTON Laurence, Versailles. BERTRAND Lionel, Montauban. BISSOLOKELE Pascal, Libourne. BRODEUR Sarah, Le Mans. BROUSSE Benoît, Elbeuf. BURGGRAFF Eric, Paris. CANAVAGGIO Pauline, Paris. CELERIER Julie, Suresnes. CHAMAA Ghassan, Evreux. CHARPENTIER Sandrine, Toulouse. CLAESSENS Yann-Erick, Monaco. CLARET Pierre-Géraud, Nîmes. COSSE Yves, Bayeux. CRETON Christophe, Prades. CURAC Sonja, Clichy. DALLE Ludovic, Versailles. DARMEDRU Amélie, Saint-Denis de la Réunion. DEBUC Erwan, Paris. DE CAGNY Bertrand, Amiens. DESMETTRE Thibaut, Besançon. DIEZ Stéphane, Kremlin-Bicêtre. DIRER Marie-Annick, Brest. DUBLANCHET Nicolas, Clermont-Ferrand. DUCHATEAU François-Xavier, Clichy. DUCHENNE Jonathan, Aurillac. DUMORA Yves, Beaumont sur Oise. DUPAS François, Pontoise. EL OMRI Mouna, Toulon. FAOUR Ali, Bernay. FERAL-PIERSSENS Anne-Laure, Paris. FIEVET-BROCHOT Marie-Laurence, Paris. FOREL Alban, Saint Julien en Genevois. FORT Pierre-Arnaud, Agen. FOUDI Lachène, Melun. FOURNIER Marc, Marseille. FRADIN Philippe, La Roche sur Yon. GAUDIN Bénédicte, Vernon. GIL JARDINE Cédric, Bordeaux. GLOAGUEN Aurélie, Dijon. GUERRIER Marion, Chartres. HAMEL Valérie, Nantes. HERNANDEZ Gilles, Moulins. HERVIEUX Aurélie, Bry/Marne. HUILLET Anne-Sophie, Cambrai. JACQUET Edouard, Foix. JAINSKY Laure, Issoire. JAVAUD Nicolas, Bondy. JOLY Luc-Marie, Rouen. KABSY Yassine, Nice. KASDALI Rachid, Beauvais. KERBAUL François, Marseille. KOUKA Clément, Saint-Denis. LABOUREY Jean-Marc, Besançon. LAPOSTOLLE Frédéric, Bobigny. LATAPPY Marc, Chambery. LAUQUE Dominique, Toulouse. LE SIRE Francis, Le Havre. LECLERCQ Olivier, Le Havre. LECONTE Philippe, Nantes. LEROY Christophe, Colombes. LESPIAUCQ Christine, Clermont-Ferrand. LOEB Thomas, Garches. MARTINEZ Mikaël, Forez. MASSOL Valérie, Dieppe. MAUSSET Véronique, Tours. MICHELET Pierre, Marseille. MILOJEVITCH Ester, Dijon. MOQUAY Romain, Valenciennes. MORIGNOT Dominique, Grenoble. OUDET Julie, Toulouse. PERIBOIS Guillaume, Thonon les Bains. POHER Fabien, Boulogne sur Mer. PRINCIPE Alexandra, Morlaix. PRUGNE Aline, Nancy. RAECKELBOOM Benoît, Dunkerque. RANAIVOZANANY Dera, Paris. REGAL Olivier, Lyon. RERBAL Djamila, Lyon. REUTER Paul-Georges, Bobigny. RICHARD Olivier, Versailles. ROCHETEAU Annabelle, Saint-Gaudens. ROORYCK François-Xavier, Paris. ROUCHY Cécile, Châteauroux. SAMPIETRO William, Tarbes. SAVIO Christophe, Le Mans. SCHOTTE Thibault, Angers. SEBILLEAU Quentin, Lille. SERRE Patrice, Bourg en Bresse. TAKI Badreddine, Fecamp. THICOIPE Michel, Bordeaux. THIRIEZ Sylvain, Roubaix. TITOMANLIO Luigi, Paris. VALLEJO Christine, Limoges. VERRAT Anne, Paris. VIALLON Alain, Saint-Etienne. VIDAL Pierre-Olivier, Marseille. VIVIEN Benoît, Paris. VRANCKX Marc, Charleroi. YAYAHOUI Layla, Créteil.
